# Improving Advanced-Line Multiple Myeloma Care: Insights and Real-World Challenges from the EMMY Study

**DOI:** 10.46989/001c.143641

**Published:** 2025-09-18

**Authors:** Titouan Cazaubiel, Olivier Decaux, Bruno Royer, Denis Caillot, Arthur Bobin, Karim Belhadj-Merzoug, Margaret Macro, Laure Vincent, Aurore Perrot, Mohamad Mohty, Lionel Karlin, Caroline Jacquet, Laurent Frenzel, Thomas Chalopin, Cécile Sonntag, Jean Fontan, Sophie Rigaudeau, Cyrille Touzeau, Hélène Demarquette, Abdelaziz Chaib, Clémence Santana, Stéphane Darre, Benoit Bareau, Ronan Garlantézec, Cyrille Hulin

**Affiliations:** 1 Department of Hematology, Hôpital Haut Lévêque, University Hospital, Pessac, France; 2 Service d’hématologie clinique, UMR U1236, CHU de Rennes, Rennes, France; 3 Immuno-hématologie, Hôpital Saint Louis, APHP, Paris, France; 4 Hématologie Clinique, CHU Dijon, Dijon, France; 5 Hématologie, CHU Poitiers, Poitiers, France; 6 Unité Fonctionnelle Hémopathies Lymphoïdes, Centre Hospitalier Universitaire Henri Mondor, Creteil, France; 7 Service d’hématologie clinique, Centre Hospitalier Universitaire de Caen, Caen, France; 8 CHU Montpellier - Hôpital St Eloi, Service d’hématologie clinique, Montpellier, France; 9 Service d’Hématologie, Centre Hospitalier Universitaire de Toulouse, Institut Universitaire du Cancer de Toulouse - Oncopole, Université de Toulouse, Toulouse, France; 10 Service d’Hématologie Clinique, Hôpital Lyon Sud, Hospices Civils de Lyon, Pierre-Bénite, France; 11 Service d’hématologie, CHU de Nancy - Hôpitaux de Brabois, Vandoeuvre les Nancy, France; 12 Service d’hématologie adulte - Responsable centre régional de traitement de l’hémophilie CRTH - Centre national de référence des mastocytoses CEREMAST, Hôpital Necker-Enfant Malade, Paris, France; 13 Service d’Hématologie et Thérapie Cellulaire, CHU Tours, Hôpital Bretonneau, Tours, France; 14 Département d’Hématologie et Oncologie, Hôpitaux Universitaires de Strasbourg, Hôpital de Hautepierre et Hôpital Civil, Strasbourg, France; 15 Service Hématologie, CHRU Jean Minjoz, Besançon, France; 16 Service d’Hématologie, CHV André Mignot, Université Versailles Saint-Quentin, France; 17 Hematology Department, University Hospital Hôtel-Dieu, Nantes, France; 18 Department of Hematology, CH, Dunkerque, France; 19 Hemato-Oncologie et Medecine Interne, Centre Hospitalier du Pays d’Aix, Aix-en-Provence, France; 20 Service Hématologie, Centre hospitalier de Valence, VALENCE, France; 21 Department of Hematology, CH Arras, Arras, France; 22 Hematology, Les Hôpitaux Privés Rennais Cesson Sévigné - Vivalto Santé, Cesson Sévigné, France; 23 Santé publique et épidémiologie, CHU de Rennes, Rennes, France; 24 Department of Hematology, Hôpital Haut Lévêque, University Hospital, Pessac, France

**Keywords:** multiple myeloma, advanced lines, real-world study, unmet medical need

## Abstract

Managing multiple myeloma (MM) patients receiving advanced (four or more) lines of treatment is a complex challenge. Therefore, real-world data are essential to better understand and address the medical need of this challenging population. We used the EMMY cohort, a French longitudinal real-world study, to describe the characteristics and outcomes of 2127 MM patients receiving advanced-line treatments between 2017 and 2020. A wide variety of treatments were used without a predominant combination showing an evolution over time. Patients exhibited median time to next treatment and overall survival ranging from 7.8 months (95% CI: 6.7-7.8) and 19.4 months (95% CI: 17.4-22.5) in Line 4 (L4) to 4.8 months (95% CI: 3.5-6) and 12.6 months (95% CI: 8.7-16.6) in L8, respectively. The EMMY study provides valuable insights into the real-world application of advanced-line treatments, demonstrating rapid disease progression and poor outcomes in these patients before the novel anti-B-cell maturation antigen (BCMA) directed therapies. These findings highlight the critical need for novel therapies in this population.

## Introduction

Multiple myeloma (MM) is a common hemopathy whose prognosis has significantly improved in the last decade. This is mainly due to the emergence of many new treatments which are more effective and better tolerated. However, despite this progress, MM patients eventually relapse and require multiple lines of treatment (LOT). Factors such as the patient’s comorbidities, previous treatments, and available options influence treatment choice. Therefore, managing patients receiving advanced LOT (four or more) remains a significant clinical challenge.^1^ Numerous clinical trials in relapsed or refractory MM (RRMM) have demonstrated the clinical benefit of new agents like bispecific T-cell engagers and chimeric antigen receptor T-cells.^2–5^ However, in the real- world, most patients with RRMM are not eligible for such trials, particularly those at more advanced lines.^6^ Within an ever-evolving landscape of MM treatments, understanding the characteristics and real-life outcomes of these advanced-line patients is essential. However, there have been few real-world studies describing this population so far. To address this, we used the real-world French study EMMY (Epidemiology of Multiple MYeloma)^7,8^ to determine the characteristics and outcomes of patients with MM receiving advanced LOT.

## Materials and Methods

EMMY is a descriptive, multicenter, non-interventional, longitudinal cohort study conducted across 73 centers affiliated with the “Intergroupe Francophone du Myélome” (IFM).^7^ It is registered in the French Health Data Hub (n° F20220518161845) and conducted in compliance with the principles of the Declaration of Helsinki. From 2017 to 2020, the study included patients who began a new treatment (irrespective of the treatment line) for symptomatic MM within a defined annual three-month period.

Patient data were extracted from medical records and collected at each center via an electronic case report form, then centralized within the study database. A scientific committee has conducted annual data quality control. Information on age, Eastern Cooperative Oncology Group performance status (ECOG PS) score, International Staging System (ISS) score, presence of high-risk cytogenetic abnormalities (t(4;14) or a del(17p) mutation assessed using fluorescence in situ hybridization [FISH] analysis), data on prior treatments and refractoriness to these were gathered for each patient enrolled in the study. Annually, selected data on myeloma treatment, response, and medical history were retrospectively updated through 2021 at the time of the analysis.

This EMMY dataset was used to generate a cohort of patients receiving advanced LOT, in which all patients starting a fourth-line of treatment (L4) or beyond, during the different data collection periods, as well as those initially enrolled on a previous line of therapy who later received L4 or beyond during follow-up, were included.

Key endpoints were time to next treatment (TTNT), progression-free survival (PFS), and overall survival (OS) defined from the time of the treatment initiation to the subsequent treatment line or death, disease progression or death, and death from any cause, respectively.

In a real-world setting, patient progression to a treatment line and response rate were assessed using a composite variable combining two complementary approaches: one based on physician judgment and documented treatment response, and another relying on monthly clinical and biochemical assessments aligned with the IMWG criteria. Progression was confirmed if either approach, or both, indicated disease progression. The best response rate was determined based on the best response identified from both approaches.

The Kaplan-Meier method was used to generate survival curves and estimate survival probabilities with their 95% confidence intervals (95% CI).

## Results

### Patient characteristics

**Figure 1.**
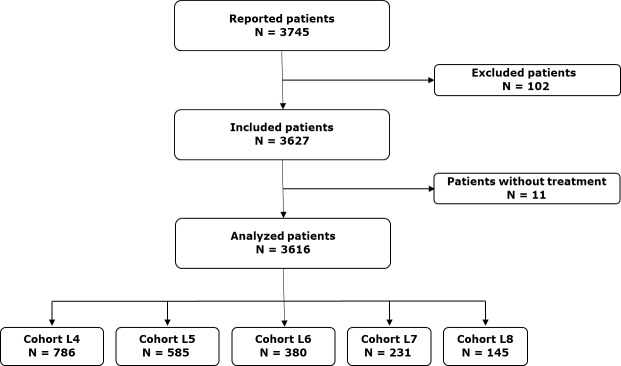
Flow-chart of the EMMY study.

From 2017 to 2020, 3616 patients were included in the EMMY study, as shown in **Figure 1**. Among these patients, a L4 or beyond was initiated 2127 times. The patient distribution was as follows: 786 patients received L4, 585 a fifth-line (L5), 380 a sixth-line (L6), 231 a seventh-line (L7), and 145 an eighth-line treatment (L8). Baseline characteristics of the patients and their disease are detailed in **Table 1**. At the initiation of L4, the median age was 72.7 years (range 27.8–94.8), and 426 patients (68.4%) had an ECOG PS score of 0 or 1. For Cohort L4, An 11-h , 35.7% of patients had high-risk cytogenetics, and 44.1% were classified as ISS stage III. The median time from diagnosis of MM to L4 was 53.9 months (range 4.3-322.6). An increasing proportion of patients with triple-class refractory (TCR) myeloma was observed across the lines: 24.4% in L4, 32.4% in L5, 55.8% in L6, 71.4% in L7, and 71.7% in L8. Similarly, refractoriness to lenalidomide and anti-CD38 monoclonal antibodies (mAbs) increased with the number of treatment lines.

**Table 1.** Patient characteristics at initiation of each line of treatment

	L4 n=786	L5 n=585	L6 n=380	L7 n=231	L8 n=145
Median age at initiation of line of interest. years	72.7 (27.8 - 94.8)	71.6 (36.2 - 95.2)	71.4 (36.4 – 96)	70.4 (44.1 - 92.4)	69.4 (45.1 - 92.6)
Median time from diagnosis to line of interest. months	53.9 (4.3 - 322.6)	67 (9.3 – 342)	80.9 (21.4 - 252.5)	91.8 (22.9 - 273.1)	103 (27 - 269.7)
ECOG PS, n (%)					
0-1	426 (68.4%)	307 (67.3%)	203 (69.8%)	122 (68.5%)	79 (66.4%)
≥2	197 (31.6%)	149 (32.7%)	88 (30.2%)	56 (31.5%)	40 (33.6%)
ISS score, n (%)					
I	133 (29.8%)	83 (26.3%)	58 (29.3%)	31 (24.8%)	21 (26.3%)
II	115 (25.7%)	75 (23.8%)	40 (20.2%)	30 (24%)	19 (23.8%)
III	197 (44.1%)	156 (49.5%)	99 (50%)	64 (51.2%)	40 (50%)
HR cytogenetics, n (%)					
Yes	116 (35.7%)	90 (35.3%)	52 (30.1%)	31 (27.7%)	15 (23.8%)
No	209 (64.3%)	165 (64.7%)	121 (69.9%)	81 (72.3%)	48 (76.2%)
Refractory status, n (%)					
Not refractory	77 (9.8%)	33 (5.6%)	9 (2.4%)	3 (1.3%)	1 (0.7%)
Lenalidomide refractory	619 (78.8%)	502 (85.8%)	339 (89.2%)	214 (92.6%)	134 (92.4%)
Anti CD38 mAb refractory	297 (37.8%)	326 (55.7%)	272 (71.6%)	189 (81.8%)	121 (83.4%)
Triple class refractory	192 (24.4%)	255 (32.4%)	212 (55.8%)	165 (71.4%)	104 (71.7%)

ISS International Staging System, HR High-risk, ECOG PS Eastern European Oncology Group Performance status, L line of treatment, mAb monoclonal antibodies.

### Treatments

Among the enrolled patients, a diverse array of treatments was utilized, such as corticosteroids, proteasome inhibitors (PIs), immunomodulatory drugs (IMiDs), alkylating agents, mAbs, B-cell maturation antigen (BCMA)-targeted therapies, and various other combinations (**Figure S1** and **Table S1**). In L4, 363 patients received treatment combinations including a PI (46.2%), 346 including an IMiD (44%), 284 including an anti-CD38 mAb (36.1%), and 196 including an alkylating agent (24.9%). The most routinely used IMiD, anti-CD38 mAb, and PI were pomalidomide (31.3%), daratumumab (30.3%), and carfilzomib (22.9%), respectively. The most frequently used treatments evolved across the LOT, with a decrease in conventional MM therapies (PIs, IMiDs, anti-CD38 mAbs) and an increase in alkylating agents and innovative treatments such as BCMA-targeted therapies and B-cell lymphoma 2 inhibitors. For example, the use of pomalidomide decreased across the lines (31.3% in L4, 19.5% in L5, 10.3% in L6, 10.8% in L7, and 5.5% in L8), whereas the use of belantamab mafodotin increased (2.7% in L4, 6% in L5, 6.6% in L6, 10.4% in L7, and 17.9% in L8). For L4 patients, the most frequently received regimens were carfilzomib-dexamethasone (10.2%), pomalidomide-dexamethasone (9.4%), daratumumab-bortezomib-dexamethasone (9%), daratumumab-dexamethasone (6.9%), isatuximab-pomalidomide-dexamethasone (5.5%), daratumumab-pomalidomide-dexamethasone (5%), and pomalidomide-cyclophosphamide-dexamethasone (4.8%). An evolution in these regimens was also observed over the years of initiation of the line of interest (**Figure S2**).

### Outcomes

In L4, the median follow-up was 5.4 months (range 0-54.4). Within this cohort of 786 patients, the overall response rate (ORR) was 50.9% with 37.3% of patients achieving a very good partial response or better and 13.7% achieving a partial response. Fifty-three patients (7.4%) maintained stable disease, while 298 (41.7%) experienced primary progressive disease. The ORR declined progressively across LOT, reflecting a diminishing treatment efficacy with subsequent regimens: 44.3% in L5, 39.2% in L6, 39.2% in L7 and 33.9% in L8.

**Figure 2.**
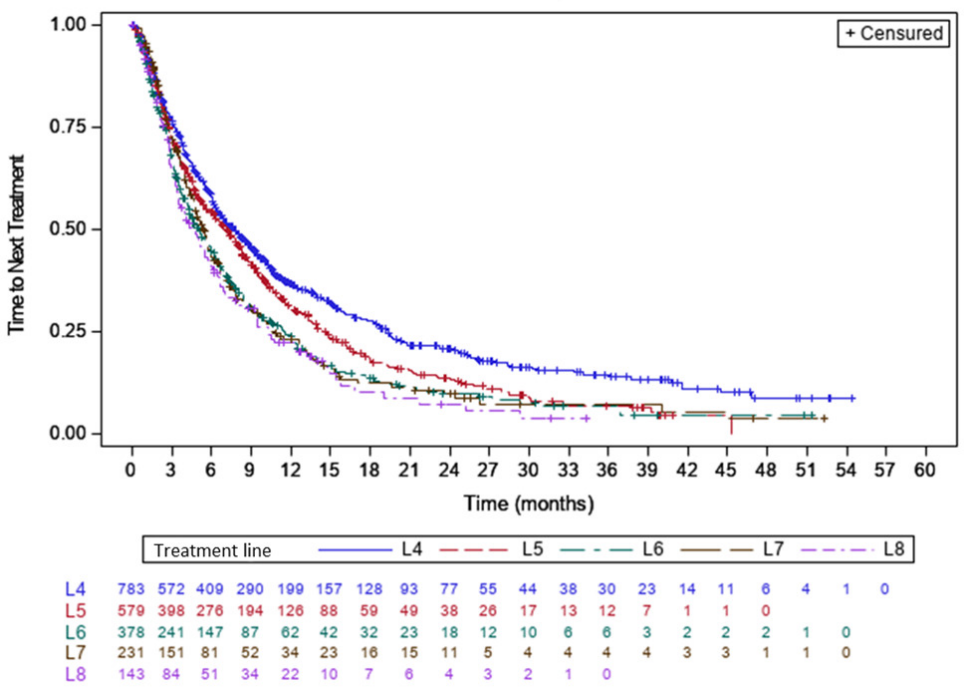
Time to Next Treatment in patients with advanced line therapy by treatment line.

Among L4 patients, 556 (70.7%) discontinued treatments, primarily due to disease progression in 377 patients (48%) and side effects in 91 (11.6%). Among L4 patients, 388 (49.4%) received subsequent therapies and the median TTNT was 7.8 months (95% CI: 6.7-7.8) (**Figure 2**). This time decreased across the LOT: 7 months (95% CI: 6-8.2) in L5, 5.1 months (95% CI: 4.3-6.1) in L6, 5.5 months (95% CI: 4.7-6) in L7, and 4.6 months (95% CI: 3.5-6) in L8.

The median PFS decreased with each subsequent line: 6.8 months (95% CI: 6-7.7) in L4, 5.8 months (95% CI: 4.8-6.6) in L5, 4 months (95% CI: 3.4-4.9) in L6, 4.7 months (95% CI: 3.9-5.3) in L7, and 4 months (95% CI: 2.8-5.3) in L8 (**Figure S3A**). The median OS was 19.4 months (95% CI: 17.4-22.5) in L4, decreasing to 15.1 months (95% CI: 13.4-16.9) in L5, 13.8 months (95% CI: 11.6-16.7) in L6, 14.5 months (95% CI: 11.3-18.7) in L7, and further reducing to 12.6 months (95% CI: 8.7-16.6) in L8 (**Figure S3B**).

**Figure 3.**
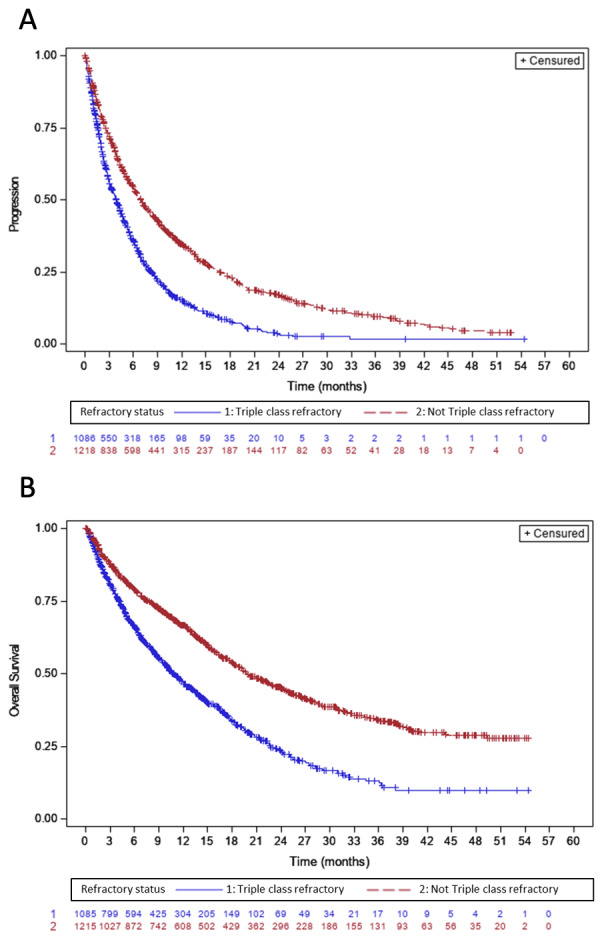
Progression free survival (A) and overall survival (B) in patients with advanced line therapy by refractory status

Refractory status had a direct impact on patient survival in advanced-line therapies. In the overall cohort, the median PFS was 3.9 months (95% CI: 3.5-4.3) for patients who were TCR, compared to 6.9 months (95% CI: 6.3-7.7) for those who were not (**Figure 3A**). Similarly, median OS was 10 months (95% CI: 9.8-12.1) and 19.9 months (95% CI: 18.3-22.4), respectively (**Figure 3B**).

## Discussion

Thanks to the growing availability of new treatments and the improved survival rates in MM, an increasing number of patients are receiving advanced LOT. The large-scale, multisite EMMY study presents a unique opportunity to gather real-world data, capturing the evolving therapeutic landscape and outcomes of over 2,000 patients undergoing L4 or beyond.

A strength of our study lies in the real-world data obtained from a large and homogeneous cohort, the patient characteristics of which were similar to other real-world analyses.^9,10^ The representativeness of our population primarily stems from the EMMY study methodology, which ensures comprehensive patient inclusion, particularly for those in advanced-line treatment. However, our population is older and frailer than those in clinical trials.^2–5,11^ For instance, in the phase 2 STORM study,^11^ evaluating selinexor for TCR MM, the median age was 65.2 years, and 88% of patients had an ECOG PS score of 0 or 1. These differences underscore the discrepancy between clinical trials and real-world populations, and the high proportion of ineligible patients in advanced treatment lines. We identified a significant proportion of patients with high-risk features (high-risk cytogenetics, ISS stage III, TCR), highlighting the complexity of managing those who face numerous relapses.

We characterized the diversity and heterogeneity of real-world advanced-line treatment patterns. Indeed, no single treatment or combination of treatments was used preferentially. Similarly, the international LocoMMotion study,^10^ a real-world, prospective study in triple-class exposed patients, reported 91 unique treatment regimens. Likewise, based on data from the French National Health Data System, Touzeau *et al*.^12^ described the treatment patterns used for 12,987 patients with RRMM between 2014 and 2018. The heterogeneity of treatments was also observed starting from L3. The diversity, heterogeneity and scalability of treatment landscapes in advanced lines emphasize the complexity of managing this population, the absence of standard-of-care therapy and the necessity of personalized, case-by-case approaches. Moreover, we observed an evolution in treatments over successive lines and years of initiation, reflecting the continuous progress in the myeloma therapeutic arsenal and its rapid impact on managing our patients in advanced-line therapy.

Like other real-world studies,^10,13–15^ we showed that advanced-line patients presented rapid disease progression, with median TTNT and PFS ranging from 7.8 months and 6.8 months in L4 to 4.8 months and 4 months in L8, respectively. Their outcomes remain poor, with median OS ranging from 19.4 months in L4 to 12.6 months in L8. Noticeably, the median PFS and TTNT in our study are very similar, underscoring the quality of the EMMY data and the accurate estimation of the incidence of disease progression in real-life settings. It is intriguing to compare our results with those of another French study^15^ in which Leleu *et al*. reported a median TTNT of 5.7 months and a median OS of 16.1 months in L4. These different results can be explained by their earlier inclusion period (from 2013 to 2017) and emphasize the permanent need for more effective treatments at later lines. Additionally, like in other reports,^16,17^ it should be noted that there are few treatment discontinuations due to side effects, despite advanced-line therapy. One possible explanation is that patients who tolerate therapy well are more likely to proceed to later LOT. Finally, similar to previous studies,^13,18^ our analysis of patient outcomes underscored the relevance of refractory status, especially for TCR patients who have a poorer prognosis, with a median OS of 10 months.

It is important to note some limitations of our study. The retrospective design explains the absence of some data, such as quality of life assessments, which remain crucial for this population. Additionally, while we reported the overall treatment discontinuation rate due to adverse effects, we could not analyze toxicity by regimen due to the heterogeneity of treatments in this population. Furthermore, the study enrollment period (from 2017 to 2020) did not include novel therapies approved since 2020, such as BCMA-targeted therapies approved in France (teclistamab, elranatamab, and idecabtagene vicleucel). On subsequent evaluations, we will be able to measure the direct impact of these therapies on the real-world management of our patients.

Overall, the EMMY study provides us a deeper understanding of the characteristics, treatment landscape, and real-world outcomes of advanced-line patients. It demonstrates the heterogeneity and rapid evolution of the treatments used, as well as the poor prognosis of this population, emphasizing a real unmet medical need. The EMMY cohort could serve as a valuable benchmark for comparison with novel therapies approved from clinical trials and for assessing their impact in real-life settings.

### Authors’ Contribution

Conceptualization: Titouan Cazaubiel, Cyrille Hulin, Olivier Decaux, Karim Belhadj-Merzoug, Margaret Macro, Aurore Perrot, Laure Vincent,; Methodology: Ronan Garlantézec, Titouan Cazaubiel, Cyrille Hulin, Olivier Decaux; Writing - original draft preparation: Titouan Cazaubiel, Cyrille Hulin, Olivier Decaux; Writing - review and editing: Titouan Cazaubiel, Cyrille Hulin, Olivier Decaux, Mohamad Mohty, Aurore Perrot, Laure Vincent, Margaret Macro, Bruno Royer, Denis Caillot, Arthur Bobin, Karim Belhadj-Merzoug, Lionel Karlin, Caroline Jacquet, Laurent Frenzel, Thomas Chalopin, Cécile Sonntag, Jean Fontan, Sophie Rigaudeau, Cyrille Touzeau, Hélène Demarquette, Abdelaziz Chaib, Clémence Santana, Stéphane Darre, Benoit Bareau, Ronan Garlantézec.

### Competing Interests

Olivier Decaux declares receiving honoraria from Bristol Myers Squib (BMS), Gilead, GlaxoSmithKline (GSK), Janssen, Roche, Sanofi, and Takeda. Denis Caillot received honoraria from BMS. Arthur Bobin received honoraria from Johnson & johnson, sanofi, amgen, pfizer, stemline menarini. Karim Belhadj-Merzoug received honoraria, research funding, and traveling expenses from Amgen, BMS, Janssen, Pfizer, Sanofi, and Takeda. Margaret Macro received honoraria and research funding from Amgen, BMS, GSK, Janssen, Sanofi, and Takeda.  Laure Vincent served on advisory board, or bibliographic presentations or writing of educational documents for Janssen, BMS, Takeda Sanofi. She received funding for congress expenses from Janssen, BMS, Sanofi, Pfizer, Takeda. Aurore Perrot served in a consulting or advisory role for Bristol Myers Squibb, Janssen, and Pfizer; and received research funding from Bristol Myers Squibb, Sanofi, and Takeda. Mohamad Mohty received honoraria, consultancy fees, and research funding from: Amgen, BMS, Gilead, GSK, Janssen, Jazz Pharmaceuticals, Novartis, Pfizer, Sanofi, Stemline Therapeutics, and Takeda. Lionel Karlin received honoraria from, served on advisory boards for, or received travel funding from AbbVie, Amgen, Janssen, Celgene/Bristol Myers Squibb, Pfizer, Sanofi, and Takeda. Laurent Frenzel received consulting fees and research funding from BioMarin, CSL Behring, Pfizer, Sobi, and Roche. Thomas Chalopin received honoraria and served in consulting role for BMS, Janssen, Sanofi, GSK, Stemline, Pfizer, Takeda. Cécile Sonntag received honoraria from, served on advisory boards for, or received travel funding from Amgen, Janssen, Celgene/Bristol Myers Squibb, Pfizer, Sanofi, and Takeda. Cyrille Touzeau served on advisory boards for and received honoraria from BMS, Amgen, Celgene, Janssen, Sanofi. Cyrille Hulin received honoraria from Janssen, Bristol Myers Squibb, Amgen, AbbVie, and Pfizer. The other authors have no competing interests to declare.

### Ethical Conduct Approval – Helsinki – IACUC

The EMMY study is classified as research not involving human subject as defined in article L. 1121-1 of the French Public Health Code. This retrospective and prospective personal data study is being conducted in accordance with the Declaration of Helsinki and has been submitted to the French Health Data (N° F20220518161845) https://www.health-data-hub.fr/projets/emmy-epidemiologie-de-laprise-en-charge-therapeutique-du-myelome-multiple-en-france. No ethical approval for this study was required. All patients were informed about the processing of their data prior to enrolment in the study.

### Informed Consent Statement

All authors and institutions have confirmed this manuscript for publication.

## Supplementary Material

Improving Advanced-Line Multiple Myeloma Care: Insights and Real-World Challenges from the EMMY Study Table S1. Treatments received by patients at each line
Figure S1 Class of drug received by patients at each line
Figure S2 Type of regimen and class of drug use in L4 depending on the year of line initiation
Figure S3 Progression free survival (A) and overall survival (B) in patients with advanced line therapy by treatment line

## Data Availability

All are available upon reasonable request.
