# Supplementary material for: Improving Advanced-Line Multiple Myeloma Care: Insights and Real-World Challenges from the EMMY Study
Source: Clin Hematol Int. 2025 Sep 18;7(3):60–8. doi: 10.46989/001c.143641 (PMC12449165; doi:10.46989/001c.143641)
Supplement: Improving Advanced-Line Multiple Myeloma Care: Insights and Real-World Challenges from the EMMY Study — Table S1. Treatments received by patients at each line Figure S1 Class of drug received by patients at each line Figure S2 Type of regimen and class of drug use in L4 depending on the year of line initiation Figure S3 Progression free survival (A) and overall survival (B) in patients with advanced line therapy by treatment line [file chi_2025_7_3_143641_299619.pdf]

*Clinical Hematology International • ISSN 2590-0048 • CHI2025*

Clinical Hematology International

# Improving Advanced-Line Multiple Myeloma Care: Insights and Real-World Challenges from the EMMY Study

**Titouan Cazaubiel et al. 2025**

**Table S1.** Treatments received by patients at each line

| | L4<br>n=786 | L5<br>n=585 | L6<br>n=380 | L7<br>n=231 |
|---|---|---|---|---|
| Glucocorticoids, n (%) | 674 (85.8%) | 461 (78.8%) | 272 (71.6%) | 160 (69.3%) |
| PI, n (%) | 363 (46.2%) | 258 (44.1%) | 167 (43.9%) | 86 (37.2%) |
| Bortezomib | 141 (17.9%) | 96 (16.4%) | 62 (16.3%) | 34 (14.7%) |
| Ixazomib | 42 (5.3%) | 38 (6.5%) | 32 (8.4%) | 15 (6.5%) |
| Carfilzomib | 180 (22.9%) | 124 (21.2%) | 73 (19.2%) | 37 (16%) |
| IMiD, n (%) | 346 (44%) | 177 (30.3%) | 89 (23.4%) | 53 (22.9%) |
| Thalidomide | 2 (0.3%) | 8 (1.4%) | 8 (2.1%) | 3 (1.3%) |
| Lenalidomide | 98 (12.5%) | 56 (9.6%) | 42 (11.1%) | 25 (10.8%) |
| Pomalidomide | 246 (31.3%) | 114 (19.5%) | 39 (10.3%) | 25 (10.8%) |
| Anti-CD38 mAb, n (%) | 284 (36.1%) | 187 (32%) | 86 (22.6%) | 34 (14.7%) |
| Daratumumab | 238 (30.3%) | 162 (27.7%) | 73 (19.2%) | 26 (11.3%) |
| Isatuximab | 46 (5.9%) | 25 (4.3%) | 13 (3.4%) | 8 (3.5%) |
| BCMA-targeted therapies, n (%) | 22 (2.8%) | 38 (6.5%) | 28 (7.4%) | 24 (10.4%) |
| CAR-T-cells | 1 (0.1%) | 3 (0.5%) | 3 (0.8%) | 0 (0%) |
| Belantamab Mafodotin | 21 (2.7%) | 35 (6%) | 25 (6.6%) | 24 (10.4%) |
| Alkylating agents, n (%) | 196 (24.9%) | 17 (30.3%) | 135 (35.5%) | 91 (39.4%) |
| Melphalan | 24 (3.1%) | 28 (4.8%) | 24 (6.3%) | 11 (4.8%) |
| Cyclophosphamide | 134 (17%) | 103 (17.6%) | 58 (15.3%) | 38 (16.5%) |
| Bendamustine | 40 (5.1%) | 54 (9.2%) | 59 (15.5%) | 44 (19%) |
| Others, n (%) | 74 (9.2%) | 92 (15.7%) | 68 (17.9%) | 48 (20.8%) |

[a] Corresponding author, Titouan Cazaubiel, e-mail: titouan.cazaubiel@gmail.com

| | | | | |
|---|---|---|---|---|
| Doxorubicine | 34 (4.3%) | 30 (5.1%) | 22 (5.8%) | 20 (8.7%) |
| Etoposide | 20 (2.5%) | 23 (3.9%) | 15 (3.9%) | 8 (3.5%) |
| Venetoclax | 21 (2.7%) | 28 (4.8%) | 28 (7.4%) | 11 (4.8%) |

BCMA B-cell maturation antigen, CAR chimeric antigen receptor, IMiD immunomodulatory drugs, mAb monoclonal antibodies, PI proteasome inhibitors.

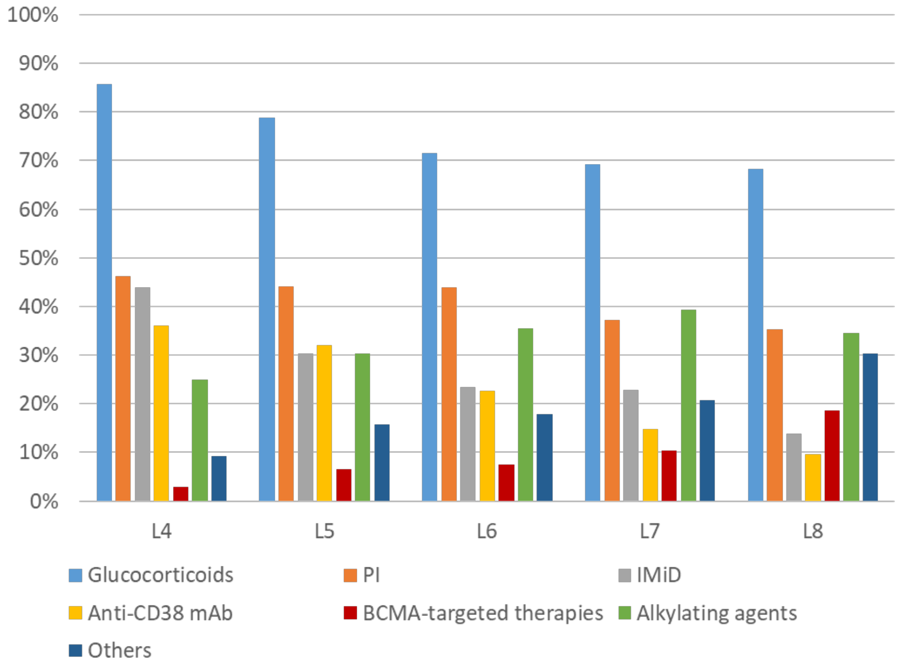


**Figure S1** Class of drug received by patients at each line

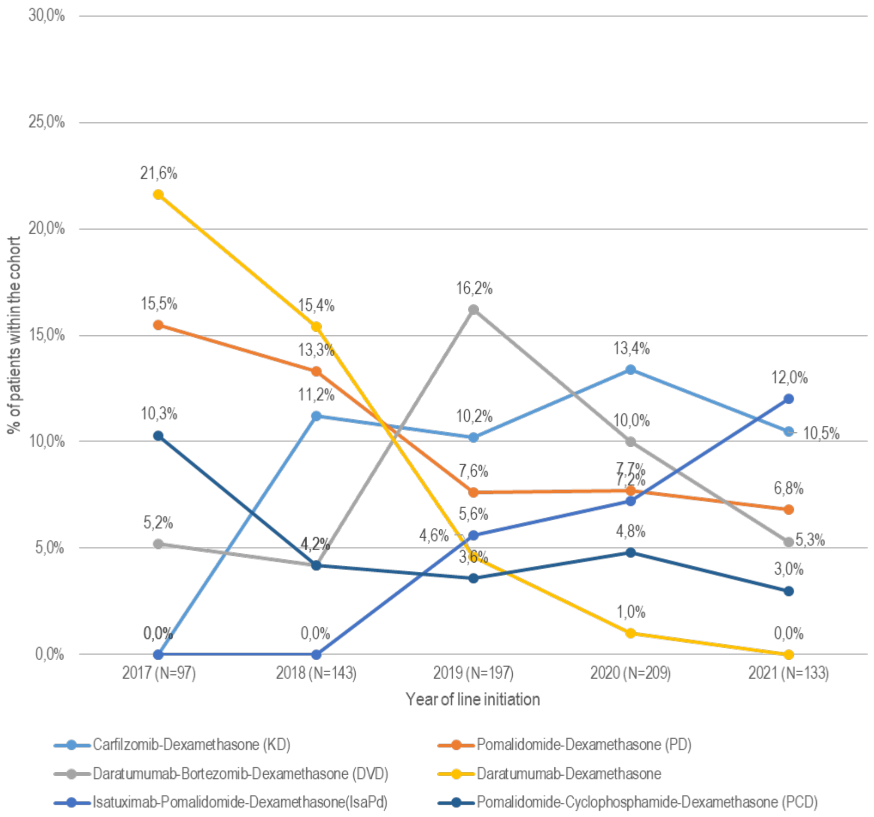


**Figure S2** Type of regimen and class of drug use in L4 depending on the year of line initiation

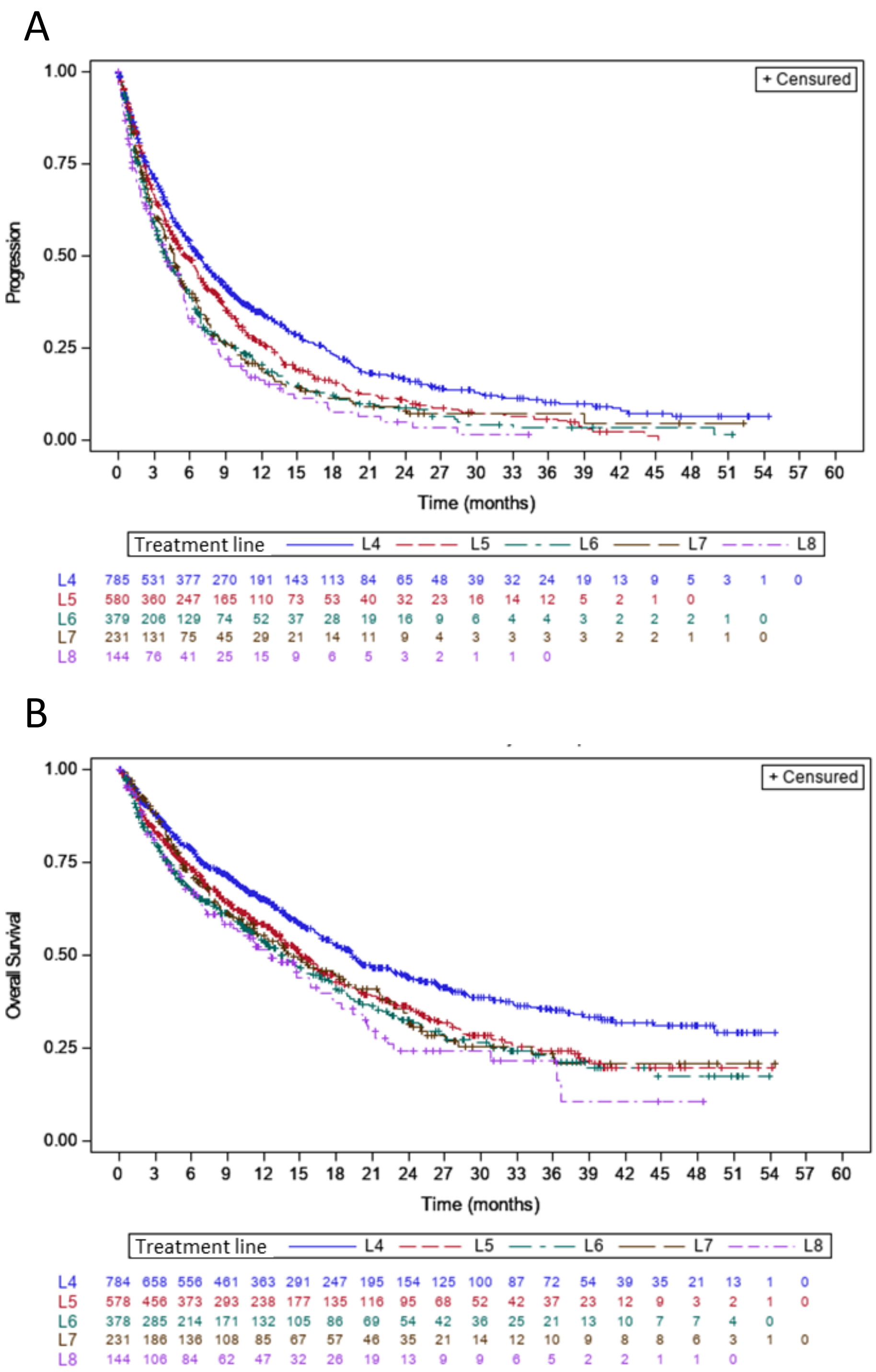


**Figure S3** Progression free survival (A) and overall survival (B) in patients with advanced line therapy by treatment line
